# Smoking Cessation Training and Treatment: Options for Cancer Centres

**DOI:** 10.3390/curroncol29040183

**Published:** 2022-03-24

**Authors:** Wayne K. deRuiter, Megan Barker, Alma Rahimi, Anna Ivanova, Laurie Zawertailo, Osnat C. Melamed, Peter Selby

**Affiliations:** 1 Nicotine Dependence Service, Centre for Addiction and Mental Health, 1025 Queen Street West, Toronto, ON M6J 1H4, Canada; wayne.deruiter@camh.ca (W.K.d.); megan.barker@camh.ca (M.B.); alma.rahimi@camh.ca (A.R.); anna.ivanova@camh.ca (A.I.); laurie.zawertailo@camh.ca (L.Z.); osnat.melamed@camh.ca (O.C.M.); 2 Dalla Lana School of Public Health, University of Toronto, 155 College Street, Toronto, ON M5T 1M7, Canada; 3 Department of Pharmacology and Toxicology, Faculty of Medicine, University of Toronto, Medical Sciences Building, Room 4207, 1 King’s College Circle, Toronto, ON M5S 1A8, Canada; 4 Department of Family and Community Medicine, University of Toronto, 500 University Ave, Toronto, ON M5G 1V7, Canada; 5 Department of Psychiatry, University of Toronto, 250 College Street, Toronto, ON M5T 1R8, Canada

**Keywords:** tobacco use disorder, smoking cessation, cancer, cancer prevention, cancer care

## Abstract

Patients who achieve smoking cessation following a cancer diagnosis can experience an improvement in treatment response and lower morbidity and mortality compared to individuals who continue to smoke. It is therefore imperative for publicly funded cancer centres to provide appropriate training and education for healthcare providers (HCP) and treatment options to support smoking cessation for their patients. However, system-, practitioner-, and patient-level barriers exist that hamper the integration of evidence-based cessation programs within publicly funded cancer centres. The integration of evidence-based smoking cessation counselling and pharmacotherapy into cancer care facilities could have a significant effect on smoking cessation and cancer treatment outcomes. The purpose of this paper is to describe the elements of a learning health system for smoking cessation, implemented and scaled up in community settings that can be adapted for ambulatory cancer clinics. The core elements include appropriate workflows enabled by technology, thereby improving both practitioner and patient experience and effectively removing practitioner-level barriers to program implementation. Integrating the smoking cessation elements of this program from primary care to cancer centres could improve smoking cessation outcomes in patients attending cancer clinics.

## 1. Introduction

Tobacco is the leading cause of preventable death, responsible for over 8 million fatalities worldwide each year, with 7 million resulting from direct use and 1.2 million from second-hand exposure [1]. In 2019, approximately 4.7 million (14.8%) Canadians aged 12 and older (17.3% male; 12.3% female) reported smoking cigarettes either daily or occasionally [2]. In the real world, practical definitions of smoking status are required to initiate medications for smoking cessation. A smoker is anyone who smoked a cigarette in the last 30 days. An ex-smoker is someone who smoked cigarettes (at least 100 cigarettes or 5 packs in their lifetime) but has not smoked—even a puff—in at least the last 6 months. Recent quitters are those that have intentionally stopped smoking for 1 day to 6 months or less [3]. Studies have established a causal connection between smoking and several cancer types, including the lung, head and neck, pancreatic, gastric, and cervical cancer [4]. Before lung or pharyngeal cancers develop, there is a high risk of respiratory infections, including pneumonia, chronic cough, bronchitis, worsening asthma, and chronic obstructive pulmonary disease (COPD) [5,6]. Various carcinogens are found in cigarette smoke, including polyaromatic hydrocarbons (PAHs), N-nitrosamines, aromatic amines, 1,3-butadiene, benzene, aldehydes, and ethylene oxide [7,8]. While nicotine is the primary addictive component of tobacco, it is not a carcinogen. However, nicotine is a cancer promoter, increases angiogenesis and inhibits apoptosis, and promotes chemotherapy resistance by creating a favourable environment for the growth and survival of mutated cancer cells [9,10,11]. Furthermore, continued tobacco use following a cancer diagnosis is related to all-cause and cancer-specific mortality, cancer recurrence, a second primary cancer, treatment-related toxicity, and an overall poorer treatment response [12]. Therefore, smoking cessation is not just primary prevention for cancer but also an important intervention for secondary and tertiary prevention in people living with cancer.

Despite the consensus that smoking cessation treatment needs to be an integral part of oncological care [13,14,15,16], approximately 40% of new patients attending Ontario cancer centres were not assessed for smoking behaviour [17]. Smoking cessation following a diagnosis of cancer can enhance clinical outcomes and reduce mortality risk by 30–40% [12,18,19,20], as well as help prevent smoking relapse [21] and second primary cancers [12]. Even in the context of healthy smokers, smoking cessation is the primary way to prevent a range of cancers, heart and lung diseases, and other chronic conditions such as diabetes [12]. For individuals who qualify for lung cancer low-dose CT (LDCT) scans, smoking cessation is an important component of the screening program [22]. Therefore, it is essential that cancer centres provide appropriate training and education for healthcare providers (HCP), as well as treatment options for smokers. This paper discusses a learning health system in smoking cessation in ambulatory community settings (i.e., Nicotine Dependence Service (NDS)). The NDS integrates research, evaluation, education, implementation, and clinical services to iteratively scale and spread smoking cessation services in Ontario, Canada. To inform our discussion, we conducted a focused literature search in addition to programmatic description and publications from the NDS. The NDS could be adapted to inform a potential model of care in cancer centres. This paper describes: (1) smoking cessation treatment options; (2) integrating smoking cessation treatment into cancer care; (3) the utility of specialized training in smoking cessation for HCP working in cancer centres (e.g., the Training Enhancement in Applied Counselling and Health (TEACH) Project)); (4) the benefit of integrating a novel model of personalized smoking cessation treatment for patients in cancer care settings (i.e., Smoking Treatment for Ontario Patients (STOP) Program model)); and (5) integrating elements of the TEACH Project and STOP Program into cancer care.

## 2. Discussion

### 2.1. Smoking Cessation Treatment Options

The majority of cancer survivors, particularly those who were currently smoking, are either unaware or disagree that the continuation of smoking is related to poorer cancer treatment outcomes [23]. These findings demonstrate a need for HCP to provide patients with educational resources and counselling on the benefits of smoking cessation after a diagnosis of cancer [23]. The 5 A’s approach represents a brief and effective clinical tool that HCP can use to identify and treat patients with evidence-based medications and counselling [24]. The 5 A’s approach involves: (1) Asking patients about their tobacco use; (2) Advising patients to quit; (3) Assessing the patient’s readiness or willingness to quit smoking; (4) Assisting in providing treatment through behavioural counselling and pharmacotherapy; and (5) Arranging follow-up visits to provide additional support [24]. A systematic review of randomized clinical trials has shown that the delivery of in-person counselling and cessation pharmacotherapy (i.e., NRT, varenicline, or bupropion) are effective in assisting individuals eligible for lung cancer screening to quit smoking 12 months post-intervention [25]. In a recent randomized controlled trial of patients diagnosed with cancer, intensive or sustained behavioural telephone counselling (i.e., four weekly sessions, four bi-weekly sessions over two months and three monthly booster sessions) in conjunction with pharmacotherapy was more effective in helping patients quit smoking when compared to standard treatment (i.e., four weekly telephone counselling meetings and medication education/advice) (34.5 vs. 21.5%) [26]. Clinician tips to assist individuals in achieving smoking cessation can be found in Table 1.

### 2.2. Integrating Smoking Cessation Treatment into Cancer Care

The Canadian Partnership Against Cancer (CPAC) provides detailed smoking cessation guidelines for oncological settings [13,30]. A crucial element of quality cancer care is the inclusion of smoking cessation behavioural counselling and referral to related treatment services using a multipronged approach [13,30,31]. Tobacco treatment should be delivered within coordinated and multidisciplinary cancer settings, whereby providers collaborate with patients and their caregivers to formulate a personalized care plan [13,31,32,33]. Furthermore, there may be some resistance among patients in initiating a smoking cessation program, and an assessment of factors that affect quit motivation, expectations around smoking abstinence, and brief motivational interventions to encourage participation in tobacco treatment should be considered as part of programming [34,35,36]. Research suggests that smokers with or without cancer histories can achieve similar abstinence rates with a comprehensive, evidence-based tobacco treatment program that incorporates pharmacotherapy and behavioural counselling [37]. For cessation treatment to become a standard practice within cancer care, it is recommended that cancer care centres incorporate tobacco screening processes into their current staff workflows, support employees’ educational needs and utilize clinicians with expertise in addiction and mental health [38,39,40,41].

### 2.3. Hcp Training: The Teach Project

Many HCP in cancer care settings do not feel adequately trained to deliver smoking cessation interventions and are uncertain where to refer their patients for more intensive and ongoing smoking cessation support [42]. Evidence has shown that HCP who receive training in smoking cessation are more likely to provide their patients with support for quitting [43,44]. Therefore, adequate training in smoking cessation for HCP is necessary to provide sufficient support with their patients who smoke.

In 2006, the NDS at the Centre for Addiction and Mental Health (CAMH; Toronto, ON, Canada) launched the TEACH Project, a comprehensive, interprofessional, and evidence-based smoking cessation training program. The goals of the TEACH Project are to (1) enhance the knowledge, skills, and confidence of HCP in the delivery of specialized, evidence-based smoking cessation interventions, and (2) increase system capacity in smoking cessation interventions. TEACH offers a university-accredited certificate program in cessation counselling, specialized courses tailored to specific practice settings and/or high-risk populations, ongoing networking and coaching from experts in the field, and follow-up professional development opportunities to HCP. All courses are delivered online to increase treatment capacity in rural and remote areas of Canada. TEACH applies best practice approaches to adult learning and interprofessional collaboration to deliver high-quality education [45]. TEACH is operationalized through the knowledge-to-action (KTA) framework, an iterative and complex process used to create and apply knowledge to practice [46].

Since 2006, TEACH has trained more than 6200 HCP and engaged 1450 unique organizations across Canada. TEACH also has engaged more than 1000 HCP, stakeholders, and tobacco control professionals in the TEACH Community of Practice, an online listserv where subscribers share and discuss clinical practice questions and cutting-edge research in tobacco control. Through the implementation of a robust evaluation framework based on Moore’s Model of Outcomes Assessment [47], TEACH has evaluated the degree to which training has affected HCP performance and patient quit rates. TEACH-trained HCP report an increase in providing smoking cessation counselling (44% prior to taking the course versus 81% at 6 months post-course, *p* < 0.001) [48] and a greater likelihood of patients quitting smoking at 6-month follow-up [40].

In 2019, TEACH partnered with CPAC to develop a series of educational modules to increase the knowledge and skills of HCP in the delivery of smoking cessation interventions in cancer care settings and support consistency in the implementation of interventions across Canada. To co-create relevant and evidence-based curriculum, TEACH collaborated with an advisory group of cancer care and smoking cessation experts across North America representative of HCP working in oncology. CPAC’s Pan-Canadian Action Framework for Implementing Smoking Cessation in Cancer Care (2019) provided the guiding framework for course development [30].

The resulting course, Tobacco Interventions in Cancer Care Settings, is composed of four standalone modules focused on key competencies in providing smoking cessation treatment. To increase access to the content and build treatment capacity across Canada, the course is delivered online at no cost to HCP who can self-enroll and revisit the content at any time. The course is also available in French to increase accessibility to Francophone Canadians. As of 1 February 2022, over 275 HCP have enrolled in the course since launching in October 2020. To access the course in English and French, visit: https://teach.camhx.ca/moodle/?redirect=0 (accessed on 23 March 2022).

In 2020, TEACH collaborated with Ontario Health to develop an online, patient-facing module to support people living with cancer. The module, Quit Smoking: For People with Cancer, outlines the benefits of quitting smoking while undergoing cancer treatment and for general health, and provides cessation strategies and resources to help people living with cancer to quit or reduce smoking. HCP working in cancer care settings are encouraged to share this module with patients and their families to support them with their quitting or reduction journey. To access the course in English and French, visit: https://elearning.cancercare.on.ca/course/index.php?categoryid=18 (accessed on 23 March 2022).

### 2.4. A Novel Model for Smoking Cessation Treatment: The Stop Program

#### 2.4.1. Rationale and Purpose

Providing access to personalized treatment and pharmacotherapy is also essential when integrating smoking cessation programs with cancer care settings [39]. However, pharmacotherapy can be expensive and is often acknowledged as a barrier to achieving smoking cessation [49,50]. When this barrier is addressed through the provision of cost-free NRT products, smoking cessation rates have been shown to increase [51,52].

In 2005, the NDS at CAMH, with funding support from the Ontario Ministry of Health, implemented the STOP Program. The goal of this initiative is to assist individuals in achieving smoking cessation, thereby reducing the prevalence of tobacco use in Ontario. This initiative is accomplished by: (1) providing access to NRT medications at no cost to the patient, thereby reducing inequities associated with access to pharmacological aids; (2) offering evidence-based training through the TEACH Project to HCP from participating STOP sites to provide smoking cessation counselling to their patients; and (3) providing ongoing capacity-building for HCP through bi-weekly conferences to support program implementation, complex clinical cases, and education in smoking cessation.

#### 2.4.2. Eligibility Criteria and Enrollment

Patients in the STOP Program are enrolled by their HCP from one of the partnering primary care settings. Eligibility criteria for the STOP Program include being a resident of Ontario and currently smoking cigarettes. Patients are enrolled in the STOP Program through a centralized online application known as the STOP Portal. Patient data is collected and shared in curated reports with affiliated primary care settings through this secure web-based portal. At enrollment, STOP implementers will complete a digital baseline questionnaire with the participant, which includes questions pertaining to the patient’s current tobacco use and dependence.

#### 2.4.3. STOP Program Model

During a 12-month enrollment period, eligible patients can receive a maximum of 26 weeks of NRT at no charge, as well as behavioural counselling. Types of NRT available to patients include patches, gum, lozenges, and inhalers. The type and amount of NRT given at each visit is determined by the practitioner in consultation with their patient. Visits occur every two to four weeks where current smoking is assessed, behavioural counselling is provided, and personalized NRT is dispensed. We understand the difficulty many people have in quitting smoking; therefore, re-enrollment into the STOP Program is permitted after the 12-month treatment period is completed, assuming the patient meets eligibility criteria at that time.

#### 2.4.4. Follow-Up Questionnaires and Primary Outcome

Patients are asked to complete a follow-up questionnaire at 3-, 6-, and 12-months following enrollment. The follow-up questionnaire is intended to assess a patient’s current tobacco use. The follow-up questionnaire is completed via the STOP Portal either by the patient via emailed survey link, with STOP staff via telephone, or with their HCP. The primary outcome of the STOP Program is 7-day point prevalence abstinence (PPA) from cigarette smoking at each follow-up period.

#### 2.4.5. Reach and Effectiveness of the STOP Program

Currently, the STOP Program is operational in 85% of Family Health Teams (FHTs), 85% of Community Health Centres (CHCs), 72% of Nurse Practitioner-Led Clinics (NPLCs), and 17% of Addiction Agencies (AAs) in the province of Ontario, with approximately 25,000 patients enrolling each year. Many STOP enrollees include individuals from subgroups of the populations who are at high risk of tobacco use and, therefore, potentially at high risk for cancer (Table 2). In the current operational models of STOP, the end of treatment and end of program follow-up smoking quit rates range from 27 to 34%. These findings indicate that a comprehensive program with the goal of training HCP and providing tobacco pharmacotherapy at no cost is an effective strategy in achieving smoking cessation.

### 2.5. Integrating Elements of Stop and Teach into Cancer Care

In addition to providing a tool for clinicians that can be used to address tobacco use and other behaviours, the STOP Portal is also used as a data collection platform to generate new knowledge that can eventually be implemented into practice in a knowledge-to-action cycle. For example, an analysis of STOP data showed that over 25,000 (approximately 35%) of STOP patients enrolled between 1 January 2014 and 21 August 2017 met the eligibility criteria for a lung cancer LDCT scan. Approximately 1300 STOP patients who met the eligibility criteria for an LDCT scan for lung cancer were randomly selected for the purpose of calculating the travel time between the patient’s residence and the closest hospital site that offered LDCT scans for high risk individuals. The results indicated that additional locations that provide LDCT scans for lung cancer are needed across the province to increase accessibility [54]. In this case, the STOP Portal was used to identify a potential barrier to access screening procedures. Another analysis of STOP data examined the quit rates and treatment characteristics of patients with self-reported cancer diagnoses compared to those patients who did not self-report a cancer diagnosis. STOP patients with a self-reported cancer diagnosis reported a 12-month follow-up PPA quit rate of 29.8%. This was comparable to the 12-month quit rate for patients without a cancer diagnosis (30.5%), suggesting that patients with cancer are as likely to quit smoking as non-cancer patients. Furthermore, during the 12 months of treatment, patients with a diagnosis of cancer demonstrated a greater duration of treatment (14.8 ± 14.8 weeks vs. 13.4 ± 14.7 weeks) when compared to their counterparts who did have a cancer diagnosis. These findings suggest that cancer centres may need to provide patients with a greater volume of resources in the form of follow-up visits to achieve cessation rates similar to patients without a cancer diagnosis.

Providing evidence-based behavioural counselling and personalized pharmacotherapy is essential for smoking cessation treatment in ambulatory cancer care facilities [24]. In the STOP Program, HCP can receive training on providing behavioural counselling and prescribing pharmacotherapy through the TEACH Project. The TEACH Project enhances the knowledge, skills, and confidence of HCP in primary care settings to provide evidence-based smoking cessation interventions. Training has shown to increase HCP engagement in smoking cessation counselling as well as patient quit rates. Integrating the TEACH HCP training modules that focus on screening for tobacco use, relapse prevention, and follow-up support into cancer facilities could be feasible and straightforward. The TEACH modules are offered online at no cost to HCP and are self-directed. Thus, these modules have the potential to remove barriers associated with accessibility and time constraints that HCP may experience with traditional training modalities (e.g., in-person, costly, or lengthy). Similar to the TEACH Community of Practice, cancer care settings could also engage HCP through an online listserv which would provide an additional opportunity to share and reconcile clinical concerns. The STOP Program provides NRT at no cost to patients. This removes the cost barrier that may hinder quit attempts, particularly among low-income individuals. The current STOP program provides behavioural counselling and access to NRT medications at no cost to the patient for up to 26 weeks in primary care settings. For some cancer facilities, offering 26 weeks of behavioural counselling and NRT might not be feasible. In this instance, cancer care settings could implement a less intensive smoking cessation model, which provides fewer weeks of NRT and counselling sessions. Prescribers on the treatment team should initiate evidence-based medications to double the chances of successful long-term quit. These include varenicline, combination NRT, bupropion, or cytisine, based on patient preference and tolerability.

The inclusion of key enablers, person-centred strategies, culturally relevant engagement, and community partnerships are also essential elements to ensure that smoking cessation treatment programs are effective [30]. Smoking cessation treatment should be personalized and consider various unique aspects of the patient’s life (i.e., demographics, culture, beliefs). Ideally, cessation programs would involve patients in the design, coordination, and implementation of the program and invite feedback from those individuals who have participated in the program [30]. Smoking cessation programs will also need to consider the cultural background of each patient and tailor their interventions accordingly to create a safe environment [30]. To enhance culturally relevant cessation interventions, the TEACH Project online modules for HCP working in cancer care settings provide strategies for working with specific populations with high tobacco use prevalence to improve cessation outcomes. Finally, developing partnerships within the community could increase smoking cessation rates [30]. The inclusion of primary care settings (e.g., FHTs, CHCs, NPLCs, AAs, mental health agencies, and Aboriginal Health Access Centres) in the treatment of smoking cessation allows the opportunity for cancer facilities to coordinate and share resources and responsibilities among HCP, resulting in a greater likelihood of patients quitting smoking [30].

The success of smoking cessation treatment is often measured by the proportion of individuals who reduce their tobacco use or achieve abstinence for a designated period. However, the effectiveness of a smoking cessation program could be measured by several additional indicators: (1) the proportion of cancer care settings that implement a smoking cessation program which includes behavioural counselling and pharmacotherapy; (2) the proportion of cancer patients who are assessed and identified as using tobacco products; (3) the proportion of individuals using tobacco products who are referred to behavioural counselling and smoking cessation pharmacotherapy by their HCP; and (4) the proportion of referred individuals who engage in a smoking cessation program composed of behavioural counselling and pharmacotherapy [30]. These indicators could be used to measure the adoption, reach, and uptake of a smoking cessation program [30].

When considering adopting elements of TEACH and STOP in cancer care, a rigid approach to implementation is likely to lead to failure. Depending on how the health care, specifically the cancer care system is structured, all or some of this model can be adapted to fit the settings. As a first step, it requires committed leadership, collaboration, and the use of implementation science methods to understand the contexts for implementation, readiness of the settings, local leadership, and practice facilitation to ensure adoption and maintenance of the program. Given the variability in capacity and motivation of settings, implementers need to start with the readiest setting and provide bespoke capacity building to those that are less able to launch. Using continuous improvement strategies, the model can be fully implemented. This requires seamless ways to capture and analyze data. A summary of practical implications for adopting elements of TEACH and STOP in cancer care can be found in Table 3.

## 3. Conclusions

This paper describes a novel model of implementation of smoking cessation treatment within primary care settings, which can be easily adapted for cancer care settings with the potential to improve smoking cessation outcomes in patients diagnosed with cancer. Potential challenges that may prevent cancer centres from integrating smoking cessation include insufficient smoking cessation training among HCP and an inability to provide personalized pharmacotherapy treatment to patients. Additional barriers may include staffing demands, perceived priorities, and role responsibilities. Both the TEACH Project and STOP Program address these challenges by providing evidence-based training to HCP and the provision of NRT at no cost to patients. The TEACH Project and STOP Program have the potential to offer many advantages to cancer care settings, and integration into such settings would likely improve health outcomes among cancer patients.

## Figures and Tables

**Table 1:** Key Clinical Tips to Assist Individuals in Achieving Smoking Cessation.

**Treatment Tips**
Tobacco use status of patients should be assessed by practitioners on a regular basis [27].
Practitioners should implement the 5 A’s approach to promote smoking cessation [24].
To efficiently assess patient motivation in quitting smoking, practitioners can ask an overall readiness question followed by the importance confidence questionnaire. If time permits, the contemplation ladder can be used [28].
Multiple forms of treatment and attempts could be necessary before smoking cessation can be achieved [27].
Practitioners should provide counselling/support along with pharmacotherapy to effectively assist patients in quitting smoking [24,27].
Practitioners should initially prescribe varenicline to their patients [29].
**Practical tips for patients**
Smoking cessation is a journey. Do not get discouraged if relapse occurs along the way.
Relapse is most likely to occur in the first 70 days. After 100 days of abstinence, the risk of relapse is low. When relapse occurs, seek treatment early. Managing stress is important. Commit to not even taking a puff of a cigarette.
Reduction is a behavioural goal, not a health goal. Reduction may assist with quitting tobacco, but cessation is the ultimate goal.
Roadblocks to smoking cessation may include stress/anxiety, boredom, and overconfidence. Problem- and emotion-focused coping could be beneficial. Finding activities to occupy hands could prevent boredom. Eliminate cues that may trigger relapse.
Coping with the loss of tobacco can be challenging. Writing down the reasons for quitting smoking and why cessation is important could be helpful in alleviating this loss.
Other behaviours (e.g., alcohol, physical inactivity, and poor diet) may obstruct smoking cessation. Identify high-risk situations and develop an action plan to prevent additional health behaviours from hindering cessation efforts.

**Table 2:** The percentage of STOP enrollments from subgroups of populations at high risk for tobacco use.

Subgroups of Population at High Risk for Tobacco Use	% of STOP Enrollments ^1^
Current/lifetime history of physical illness (not including cancer) ^2^	57.1
Current/lifetime history of cancer	8.5
Current/lifetime history of mental illness ^3^	57.2
Hazardous levels of alcohol use in the past 30 days ^4^	32.8
Medical/recreational cannabis use in the past 30 days	31.2
Medical/recreational opioid use in the past 30 days	16.0

Note: All health conditions and substance use are self-reported. ^1^ Sample includes all eligible enrollments into the STOP program between 1 January 2014 and 31 October 2021; ^2^ Includes self-reported history of high blood pressure, heart disease, high cholesterol, diabetes, or chronic bronchitis, emphysema, COPD; ^3^ Includes self-reported history of depression, anxiety, bipolar disorder, or schizophrenia; ^4^ As per Alcohol Use Disorders Identification Test (AUDIT-C) score [53].

**Table 3:** Practical Implications for Adopting Elements of TEACH and STOP in Cancer Care.

Practical Implications by Stakeholder
**For Patients:** access to no cost evidence-based smoking cessation based on NRT that balances patient preferences with the best chances of quitting smoking may improve survival from cancer and quality of life.
**For Practitioners:** a validated care pathway with embedded decision support systems, practice facilitation, community of practice, and quality indicators maximize the opportunities for high-quality care. It can also help identify co-morbidities and sub-populations in need of additional interventions and/or referrals.
**For Clinic Managers:** process and outcome measures for quality improvement and reporting purposes, resource allocation, especially task shifting, given the automation of the care pathway that can lead to increased clinic efficiency.
**For Funders:** accountability and impact on the number one cause of preventable mortality. In patients living with cancer, this can lead to improved outcomes, less relapse, and, in certain cases, cost savings.
**For Researchers:** real-world standardized data collection and ability to link to other clinical and administrative data sets. It can also provide a stable platform for randomized trials and data access/audit.

## Data Availability

The data presented in this study are available upon reasonable request from the corresponding author. Requests for access to anonymized patient-level data will be considered in consultation with the supervising privacy and ethics bodies.
